# Lung ultrasound score ≥ 6 predicts surfactant administration decisions in meconium aspiration syndrome: a multicenter prospective study

**DOI:** 10.1016/j.jped.2026.101529

**Published:** 2026-03-20

**Authors:** Qi Chen, Zekai Yu, Lei Cao, Wei Xiong, Mei Zheng, Fei Wang, Shasha Wu, Rijin Yu, Minli Zhou, Cheng Guo, Lin Dong, Shuang Liu

**Affiliations:** a Shangrao Children's Hospital, Shangrao Maternal and Child Health Hospital, Neonatal Intensive Care Unit, Department of Neonatology, Shangrao, China; b Hangzhou Dianzi University, School of Computer Science and Technology, Hangzhou, China; c Jingdezhen First People's Hospital, Department of Pediatrics, Jingdezhen, China; d Hubei University of Medicine, Xiangyang NO.1 People's Hospital, Department of Pediatrics, Xiangyang, China; e Qinghai Red Cross Hospital, Department of Neonatology, Qinghai, China; f Yangzhou Maternal and Child Health Hospital, Department of Neonatology, Yangzhou City, China; g Panzhou Second People's Hospital, Department of Neonatology, Panzhou City, Liupanshui, China; h Jiangxi Provincial Children's Hospital, Department of Neonatal Intensive Care Unit, Jiangxi Province, China; i Affiliated Hospital of Jining Medical University, Neonatology Department, Jining City, China; j Huanggang Central Hospital, Neonatal Intensive Care Unit, Hubei Province, Huanggang City, China; k Tibet Fukang Hospital, Department of Neonatology, Luobulinka Road, Tibet Autonomous Region, China

**Keywords:** Neonatal meconium aspiration syndrome, Lung ultrasound, Surfactant, Neonatal intensive care unit, Lung ultrasound score

## Abstract

**Objective:**

Meconium aspiration syndrome is a common cause of severe respiratory failure in term and post-term neonates. The timing for administering surfactant remains non-standardized. This study aimed to evaluate the predictive value of the lung ultrasound score for the need for surfactant therapy in infants with Meconium aspiration syndrome.

**Method:**

This prospective multicenter study enrolled 218 neonates with meconium aspiration syndrome. Lung ultrasound was performed within 30 min of admission using a six-zone scoring system (0–18). Surfactant was given based on oxygenation criteria (FiO₂ > 0.5 or OI > 8), with clinicians blinded to ultrasound findings. Predictive performance of the lung ultrasound score for surfactant need was assessed by ROC analysis and compared with traditional indices. Multivariable regression and decision curve analysis were performed.

**Results:**

Lung ultrasound score demonstrated near-perfect diagnostic accuracy for predicting surfactant need: at the optimal cutoff of 6 points, AUC was 0.999 (95% CI, 0.997–1), with 100% sensitivity. Performance was superior to pH (AUC 0.906) and chest radiograph grade (AUC 0.539), and consistent across subgroups by respiratory support, gestational age, and presence of persistent pulmonary hypertension. Multivariable analysis identified lung ultrasound as an independent predictor of surfactant use. Decision curve analysis confirmed greater net clinical benefit for lung ultrasound-based strategies over a wide range of threshold probabilities.

**Conclusion:**

A lung ultrasound score of ≥ 6 exhibited near-perfect predictive capacity for guiding surfactant administration decisions (AUC 0.999). This technique provides rapid, non-invasive pulmonary morphological information, facilitating the identification of neonates meeting the criteria for surfactant therapy.

## Introduction

Meconium aspiration syndrome (MAS) is a common cause of severe respiratory failure in term and post-term neonates [1]. Its pathophysiology is complex and includes mechanical airway obstruction caused by meconium particles, chemically induced inflammatory injury to the pulmonary vasculature, and direct inactivation of surfactant by meconium components. Surfactant replacement therapy is an important treatment for moderate-to-severe MAS and can improve lung compliance and oxygenation [2,3]. However, there is currently no consensus on the indications and timing for surfactant administration. Clinical decisions largely rely on parameters such as the oxygenation index (OI) or the fraction of inspired oxygen (FiO₂) [4., 5., 6.]. These indices not only require invasive arterial blood sampling but, more importantly, reflect overall systemic oxygenation rather than directly and regionally assessing the morphological severity of lung pathology.

Lung ultrasound (LUS), a non-invasive imaging modality that can be performed at the bedside in real time, offers a new perspective for assessing pulmonary disease [7]. Technically, LUS diagnoses lung disorders by interpreting acoustic artifacts (e.g., A-lines, B-lines) and direct signs (e.g., consolidation, air bronchograms, pleural effusion) [8]. A semi-quantitative, region-based LUS scoring system objectively quantifies disease severity by dividing the lungs into predefined zones and grading the ultrasonographic findings in each zone. This scoring system has been validated for predicting surfactant requirements in neonatal respiratory distress syndrome (RDS) [9., 10., 11., 12., 13., 14., 15., 16.]. However, MAS is characterized by marked heterogeneity of lung involvement, in contrast to the diffuse changes seen in RDS; therefore, the value of LUS scoring in MAS requires independent validation.

In this prospective observational study, the authors the authors technically evaluate the ability of the LUS score to predict the need for surfactant therapy in infants with MAS. The authors The authors used the six-zone method recommended by international expert consensus — dividing each hemithorax into upper and lower regions along the parasternal, anterior axillary, and posterior axillary lines. Trained physicians performed standardized scans within 30 min of admission, and a validated scoring system (0–3 per zone; total 18) was applied. By comparing LUS with conventional oxygenation parameters (OI and FiO₂), which served as the gold-standard basis for therapeutic decision-making, the authors the authors systematically assessed predictive accuracy, the optimal diagnostic cutoff, and clinical utility to determine the potential of LUS as a non-invasive decision-support tool for optimizing surfactant treatment decisions in MAS.

## Methods

### Study design and participants

This was a prospective, observational, multicenter study conducted in two academic tertiary neonatal intensive care units (NICUs). The protocol was approved by the institutional review boards at each site, and written informed consent was obtained from the legal guardians of all infants. This study has been approved by the Ethics Committee of Shangrao Maternal and Child Health Hospital, with the ethical approval number SRFB20231206037, dated December 6, 2023.

Eligible participants were term or post-term neonates (gestational age 37–42 weeks) admitted between 2019 and 2025. Inclusion criteria were: (1) grade III meconium-stained amniotic fluid [17]; (2) postnatal respiratory distress requiring NICU respiratory support (noninvasive ventilation or invasive mechanical ventilation); and (3) clinical diagnosis of MAS. Exclusion criteria were: (1) healthy rooming-in newborns not requiring NICU care; (2) major congenital disorders or severe congenital malformations; and (3) congenital lung disease.

### Sample size and statistical power

Based on pilot data, an a priori sample size calculation was performed using G*Power 3.1. With α = 0.05, power (1−β) = 0.80, an anticipated AUC of 0.98 for the LUS score, and a null AUC of 0.90, the minimum required sample size was 156. Allowing for a 20 % loss to follow-up or incomplete data, the authors the authors planned to enroll 200 infants. Ultimately, 218 infants were included, meeting and exceeding power requirements.

### LUS examination and scoring

Timing of Lung Ultrasound Examination: Bedside lung ultrasound was performed immediately upon admission to the NICU, with a median time of 10 min from admission to completion of the lung ultrasound (The maximum duration does not exceed 30 min). This rapid assessment captured the infant's pulmonary status prior to any major therapeutic interventions beyond initial stabilization. The FiO₂ value recorded reflected the fraction of inspired oxygen required at the exact moment of the lung ultrasound examination. Surfactant administration was always performed after the completion of the baseline assessment. The median time from admission to surfactant administration was 1 hour. To ensure timeliness, each center guaranteed 24-hour on-site coverage by at least one qualified ultrasonographer. Examinations used a Mindray M9 portable system, preferentially with a high-frequency linear probe (L12–4 s), switching to a low-frequency convex probe (C 11-3s) when greater penetration was required. Dynamic cine loops were archived for subsequent analysis. Lung zoning followed recent international expert consensus, using the anterior and posterior axillary lines to divide each hemithorax, for a total of ten neonatal-specific assessment zones across both lungs, accommodating the heterogeneous distribution typical of MAS (The protocol actually used in practice was the 6-zone method) [18,19].

MAS-specific LUS scoring criteria (Brat-based system adapted to MAS pathology)

0: normal ventilation with lung sliding and A-lines or < 3 isolated B-lines;
1: moderate reduction in ventilation with ≥ 3 B-lines and/or small focal consolidation;
2: severe reduction with confluent B-lines or larger consolidation involving more than two zones in aggregate;
3: extensive consolidation with air bronchograms and/or atelectasis, tissue-like pattern.

Each zone was scored 0–3 (total 0–18). All anonymized LUS cine loops were stored on a secure server and were independently scored by two experienced raters who were blinded to all clinical information, including the infant's respiratory support level, FiO₂ requirements, blood gas results, and whether surfactant was eventually administered. Discrepancies between raters were resolved by consensus or by a third senior reviewer. Inter-rater reliability was assessed on a random subset of 30 infants with double-blind rescoring, yielding a Fleiss’ kappa of 0.85 (95 % CI, 0.76–0.94), indicating high reproducibility. To ensure objectivity, the LUS examinations were performed by a dedicated team of trained sonographers who were not involved in the clinical management of the infants. The results of the LUS examination, including the real-time images and the subsequent scoring, were not disclosed to the attending clinical team responsible for making decisions regarding respiratory support and surfactant administration.

### Clinical management and data collection

Surfactant administration followed institutional standards: among infants requiring respiratory support, treatment was given when FiO₂ > 0.5 and/or OI > 8 [20]. Crucially, as per the study protocol, the attending physicians were strictly prohibited from viewing the LUS images or scores. All decisions regarding surfactant administration were made exclusively based on the pre-defined institutional criteria (FiO₂ > 0.5 and/or OI > 8) and the infant's overall clinical assessment, without any influence from the research LUS data. This rigorous separation ensured that the predictive performance of LUS could be evaluated against an independent, real-world clinical gold standard. All surfactant was delivered via standard endotracheal instillation.

Collected data included demographics, perinatal characteristics, respiratory support modes and parameters, arterial blood gas results, surfactant use (including timing of the first dose), complications (e.g., PPHN, ECMO), and hospital outcomes. PPHN was diagnosed by echocardiographic evidence of right-to-left or bidirectional shunting in conjunction with clinical criteria.

### Statistical analysis

All analyses were conducted in R (version 4.5.1). Two-sided *p* < 0.05 was considered statistically significant.

Data preprocessing and grouping: Prior to analysis, the raw dataset underwent comprehensive cleaning and preprocessing. Missingness was handled via complete-case analysis using the dplyr and tidyr packages, and outliers were screened and addressed. The authors The authors then defined the following analytic groups: Primary comparison groups—surfactant vs. no-surfactant—used for baseline comparisons and diagnostic performance evaluation. LUS strata—LUS < 6 vs. LUS ≥ 6 based on the ROC-derived optimal cutoff—to examine management differences across score ranges. Prespecified subsets for robustness analyses included: by support type (NIV vs. IMV); by disease severity (LUS 0–5 mild, 6–12 moderate, > 12 severe); by complication status (PPHN absent vs. present); by gestational age (37–39 vs. 40–42 weeks); and by risk tier (low: LUS 〈 6; intermediate: LUS 6–10; high: LUS 〉 10).

Descriptive statistics and between-group comparisons: Normality of continuous variables was assessed using the Shapiro–Wilk test. Normally distributed data are reported as mean ± SD and compared with Student’s *t*-tests; non-normal data are reported as median (IQR) and compared with Mann–Whitney U tests. Categorical variables are presented as counts and percentages and compared using chi-square tests or Fisher’s exact tests (Table 1).

**Table 1:** Baseline characteristics.

Independent variable		No-PS	PS	P
Gestational (week)		39 (38, 41)	40 (38, 41)	0.704
LUS-score		4 (3, 5)	8.5 (7, 10)	<0.001
Oxygen-concentration		30 (26.25, 45)	50 (50, 56.25)	<0.001
pH		7.21 (7.16, 7.27)	7.08 (6.99, 7.14)	<0.001
Oxygenation-index		7 (6, 7)	10 (9, 10)	<0.001
Hospital-days		6 (5, 7)	8 (7, 9)	<0.001
Gender	Females (0)	51 (37 %)	29 (36.2 %)	1
	Males (1)	87 (63 %)	51 (63.7 %)	
X-ray-grade	1	2 (1.4 %)	0 (0 %)	0.413
	2	75 (54.3 %)	39 (48.8 %)	
	3	61 (44.2 %)	41 (51.2 %)	
Complications	NO	138 (100 %)	78 (97.5 %)	0.134
	YES	0 (0 %)	2 (2.5 %)	

**Figure 1:**
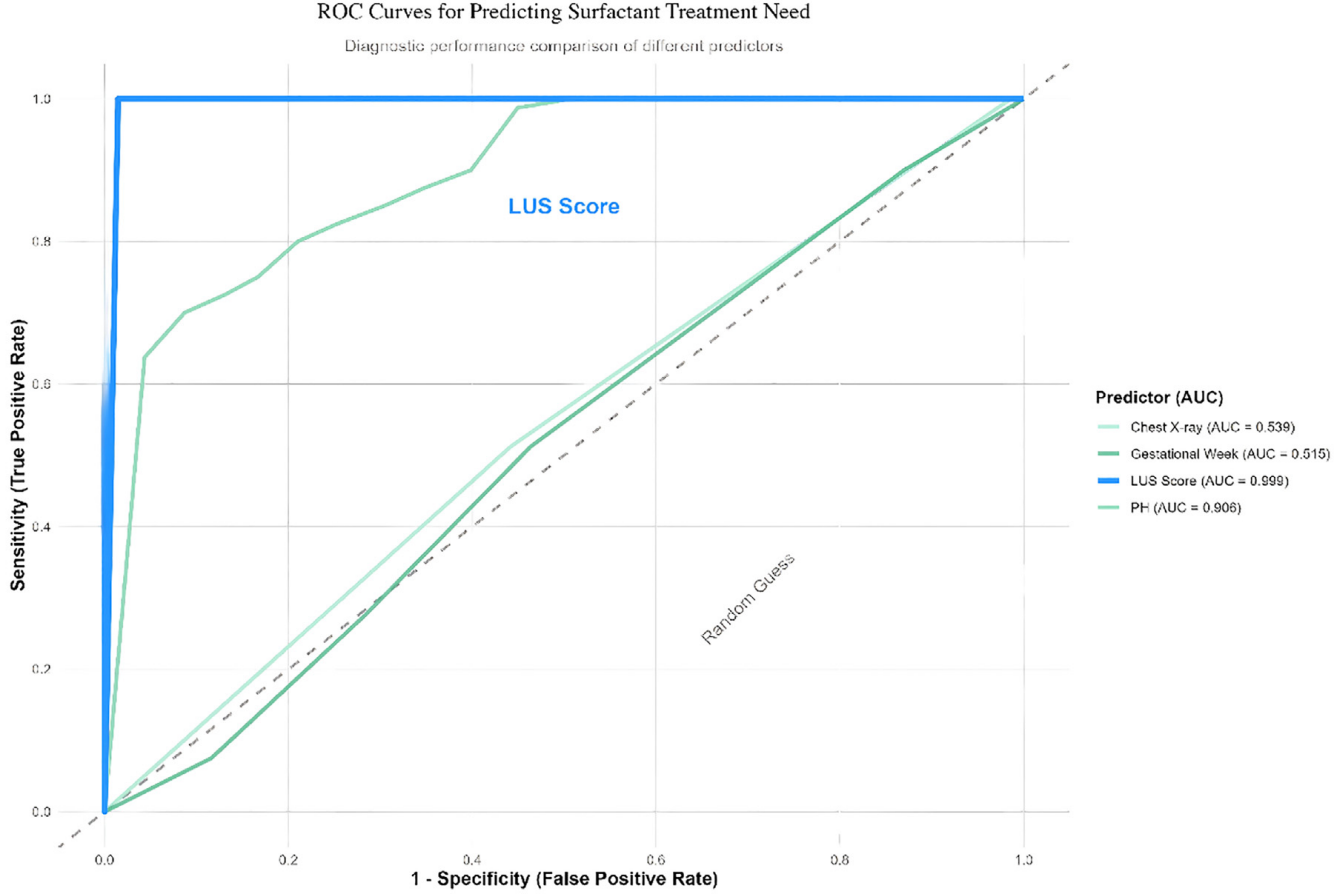
ROC curves of LUS and other variables.

Diagnostic accuracy and comparisons: ROC analyses were performed for LUS score, OI, FiO₂, and pH to predict surfactant requirement. The authors calculated AUCs with 95 % CIs. Because OI/FiO₂ defines the standard for surfactant decision-making, the authors used bootstrap resampling (1000 iterations) for robust AUC estimation and applied DeLong tests to compare LUS AUCs with traditional indices. The optimal LUS cutoff was determined by the maximum Youden index, with corresponding sensitivity, specificity, PPV, and NPV reported (Table 2; Figure 1).

**Table 2:** Diagnostic accuracy metrics.

Indicator	AUC	Sensitivity	Specificity	Accuracy	PPV	NPV
Lung Ultrasound Score (LUS) (threshold: 6)	0.999 (0.997–1)	1 (0.71–0.891)	0.812 (0.974–1)	0.931 (0.889–0.961)	0.902 (0.945–1)	1 (0.843–0.944)
pH (threshold: 7.14)	0.906 (0.868–0.945)	0.167 (0.16–0.359)	0.25 (0.109–0.24)	0.197 (0.147–0.256)	0.277 (0.093–0.219)	0.148 (0.184–0.386)
X-Ray Grade (threshold: 2.5)	0.539 (0.47–0.608)	0.558 (0.398–0.626)	0.512 (0.471–0.642)	0.541 (0.473–0.609)	0.664 (0.306–0.504)	0.402 (0.57–0.749)

Multivariable logistic regression and confounding control: To independently assess the association between LUS score and surfactant use while addressing potential confounding, the authors fit multivariable logistic regression models with surfactant use as the dependent variable. Prior to modeling, collinearity among continuous covariates was evaluated using variance inflation factors (all VIFs < 2.0). The final model included the LUS score (continuous) adjusted for gestational age, birth weight, and PPHN. Results are presented as adjusted odds ratios (ORs) with 95 % CIs (Table 3).

**Table 3:** Multivariable logistic regression analysis (adjusted for confounding bias).

Program A: Clinical Grouping				
Program A Regression Results				
Variable	Coefficient	OR	OR_CI_Lower	OR_CI_Upper
risk tier (intermediate)	−0.084759871	0.9187329	0.8095726	1.066629
risk tier (high)	0.853738226	2.3484093	1.4867197	2.629339
Gestational (week)	0.026567930	1.0269240	0.7903164	1.275278
Males(1)females(0)	0.004536994	1.0045473	0.7888395	1.269091

Program B: Winsorization				
Program B Regression Results				
Variable	Coefficient	OR	OR_CI_Lower	OR_CI_Upper
LUS-score	0.25780902	1.294092	0.9845084	1.549647
Gestational (week)	0.03086160	1.031343	0.7378134	1.387159
FiO2	0.08903672	1.093121	0.5793027	2.001482

Program C: L1 Regularization Selection				
Program C Regression Results				
Variable	Coefficient	OR	OR_CI_Lower	OR_CI_Upper
LUS-score	1.0047917	2.7313383	1.0000000	3.814383
Gestational (week)	0.0000000	1.0000000	0.7130925	1.036248
FiO2	0.0000000	1.0000000	0.4433724	5.477255
Males (1)				
Females (0)	0.0000000	1.0000000	0.8836486	1.164922
X-ray-grade	0.0000000	1.0000000	1.0000000	1.101355
pH	−0.1143144	0.8919775	0.6235915	1.000000
Model AUC Comparison				
AUC Values for Each Program				
	**AUC**			
Program A	0.9850746			
Program B	1.0000000			
Program C	1.0000000			

Interpretation.

The analysis used multivariable logistic regression models to adjust for potential confounding factors, including gestational age, FiO2, gender, etc., to more accurately assess the impact of each variable on the outcome.

OR (Odds Ratio) indicates the degree of influence of an exposure factor on the probability of the outcome:.

- OR > 1: The factor is a risk factor, increasing the probability of the outcome.

- OR < 1: The factor is a protective factor, decreasing the probability of the outcome.

- OR = 1: The factor has no significant effect on the outcome.

All models controlled for the aforementioned confounding variables, so the reported OR values are adjusted estimates.

The model AUC values indicate excellent predictive ability for all models (AUC > 0.98).

**Figure 2:**
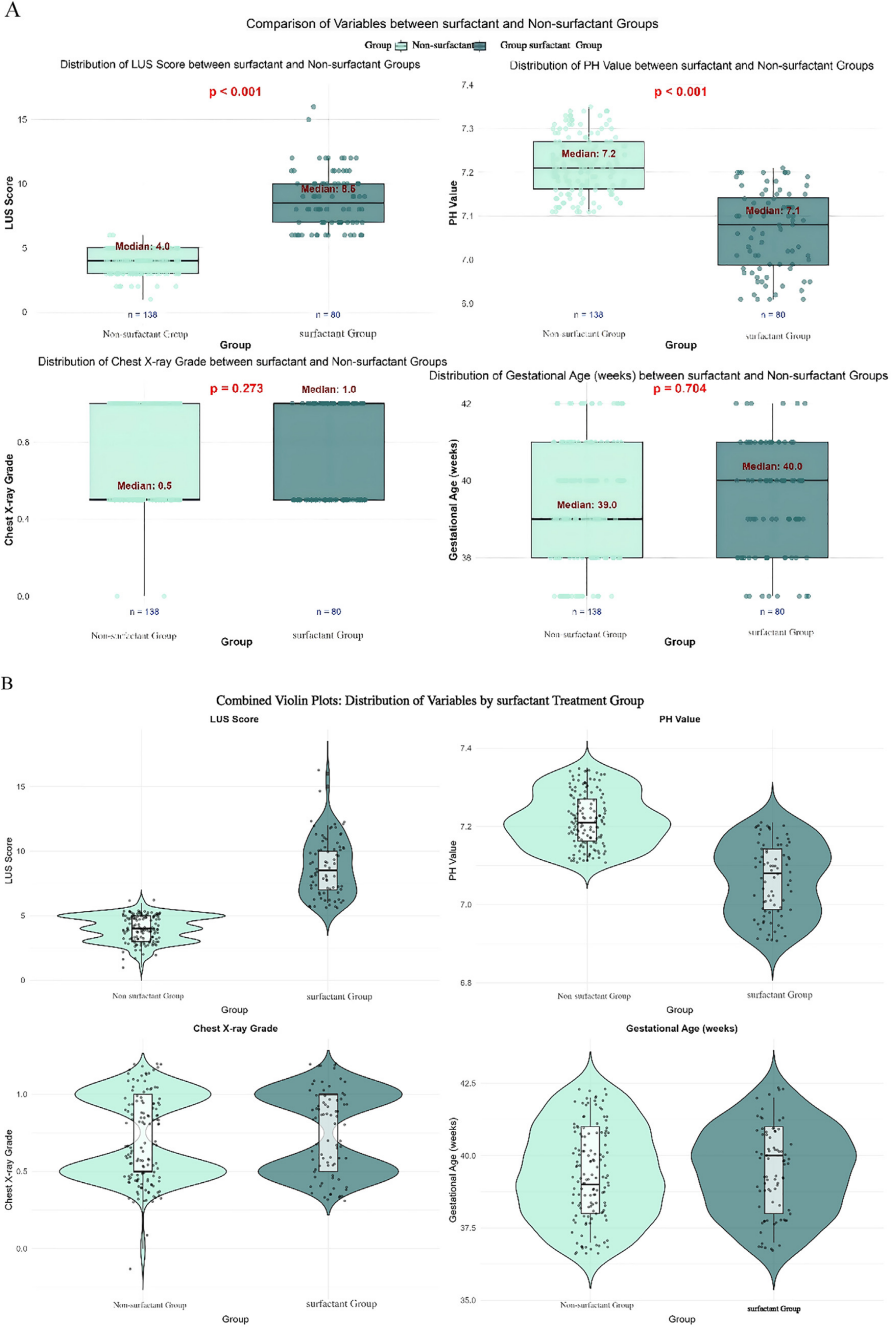
(A) comparison of variables between surfactant groups (Boxplots). (B) Variable distributions are visualized using violin plots, with embedded boxplots displaying the median, interquartile range, and outliers. - The "width" of the violin plot represents data density, while the boxplot intuitively illustrates central tendency and dispersion.

**Figure 3:**
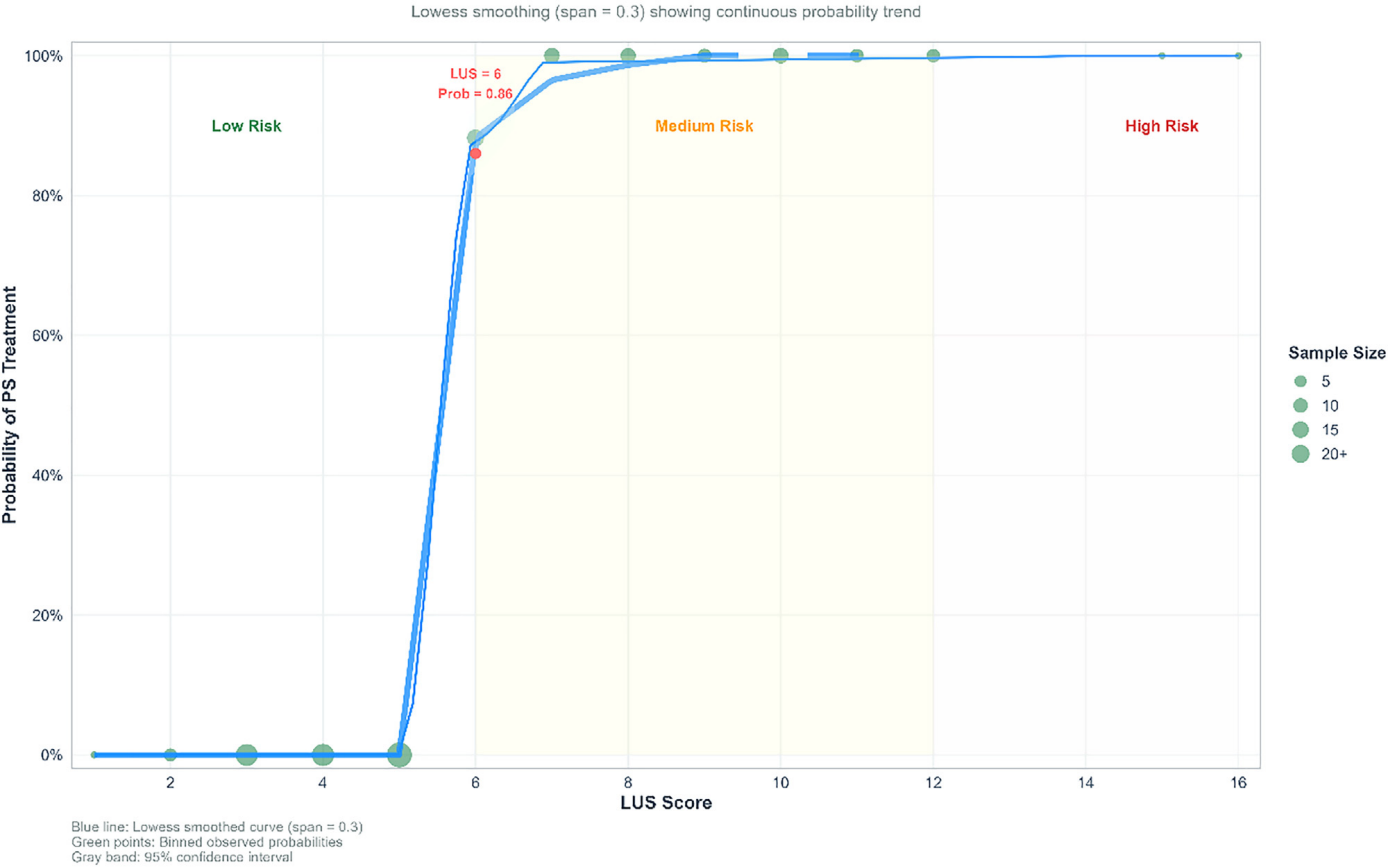
Probability curve. - risk stratification: low risk (LUS < 6): very low probability of surfactant therapy (< 10 %). - Intermediate risk (6 ≤ LUS < 12): Probability increases rapidly, approaching 100 %. - High risk (LUS ≥ 12): probability stabilizes at 100 %.

Model performance, calibration, and clinical utility:

Visualization and distribution: To visualize LUS distributions in surfactant vs. no-surfactant groups and depict overall dispersion with key statistics, the authors generated combined box-and-violin plots (Figure 2). To display the continuous relationship between LUS and the probability of surfactant use, the authors plotted restricted cubic spline (RCS) curves (Figure 3).

**Figure 4:**
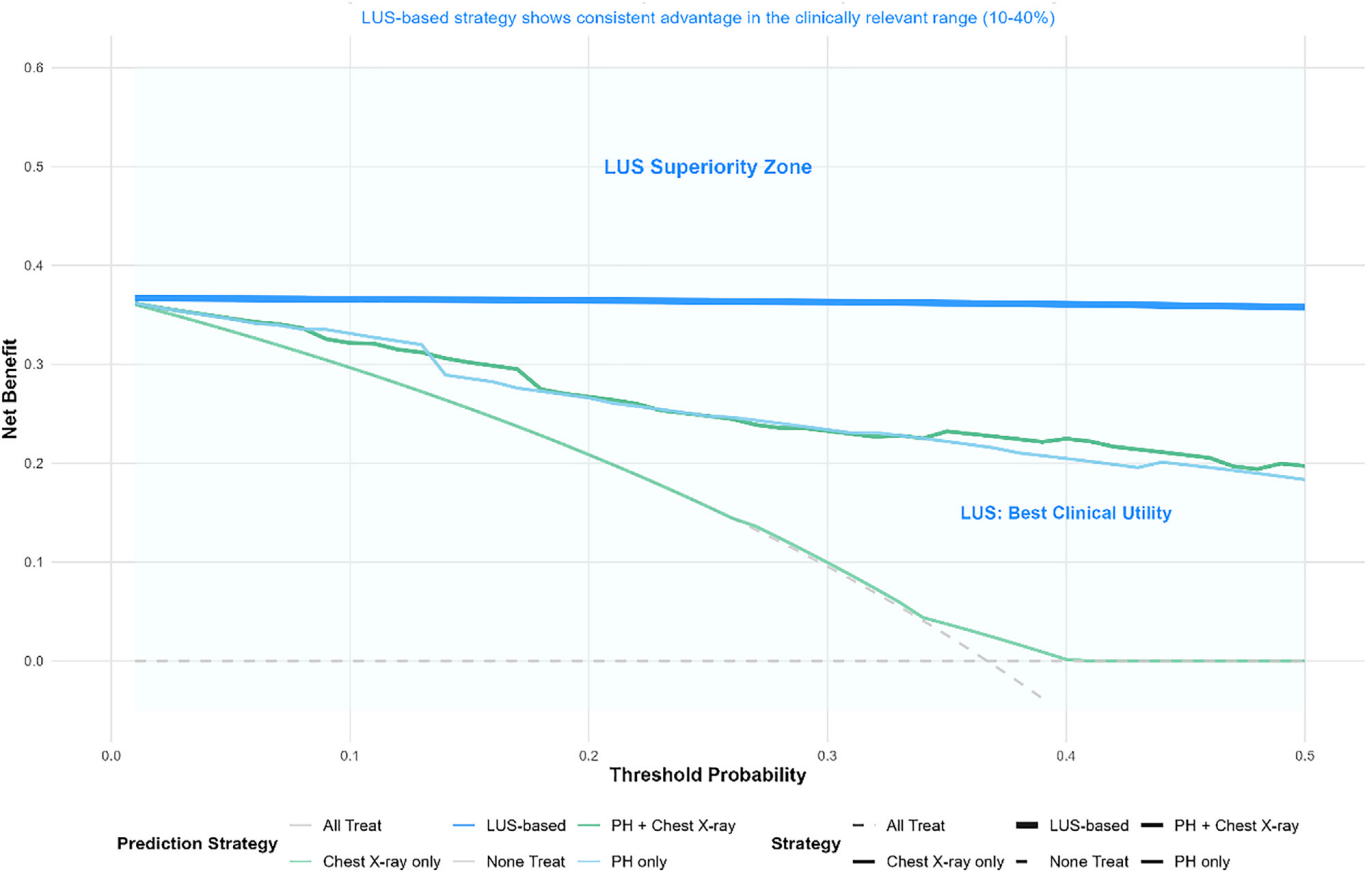
Decision curve analysis demonstrating the superiority of the LUS-based strategy: within the clinically relevant threshold range (10–40 %), the LUS-based prediction strategy yielded the highest net benefit, significantly outperforming other strategies, including pH + Chest X-ray, Chest X-ray only, and pH only.
— Calibration: The authors assessed agreement between predicted and observed probabilities using decile grouping with LOWESS smoothing (Table 5, Figure 5).

— Decision curve analysis (DCA): Net clinical benefit across threshold probabilities was compared among four strategies: (1) treat-all; (2) treat-none; (3) LUS-guided treatment; and (4) treatment guided by pH combined with chest X-ray grade (Table 4, Figure 4).

**Table 4:** Decision curve analysis - net benefits at key threshold probabilities.

Threshold Probability	All Treat Net Benefit	None Treat Net Benefit	pH Only Net Benefit	Chest X-ray Only Net Benefit	pH + *X*-ray Net Benefit	LUS-based Net Benefit	LUS Advantage vs Combined	Best Strategy
0.1	0.2966	0	0.3313	0.2966	0.3216	0.3660	**0.0443**	**LUS-based**
0.2	0.2087	0	0.2661	0.2087	0.2672	0.3647	**0.0975**	**LUS-based**
0.3	0.0957	0	0.2339	0.0996	0.2326	0.3630	**0.1304**	**LUS-based**
0.4	−0.0550	0	0.2049	0.0015	0.2248	0.3609	**0.1361**	**LUS-based**

**Table 5:** Calibration statistics for different predictors.

Predictor	Calibration Error	Brier Score	Observations
LUS Score	0.0162	0.0081	218
Chest X-ray Grade	0.4617	0.2309	218
pH	0.2259	0.1151	218
Gestational Week	0.4644	0.2322	218

Note: Lower values indicate better calibration and prediction performance.

**Figure 5:**
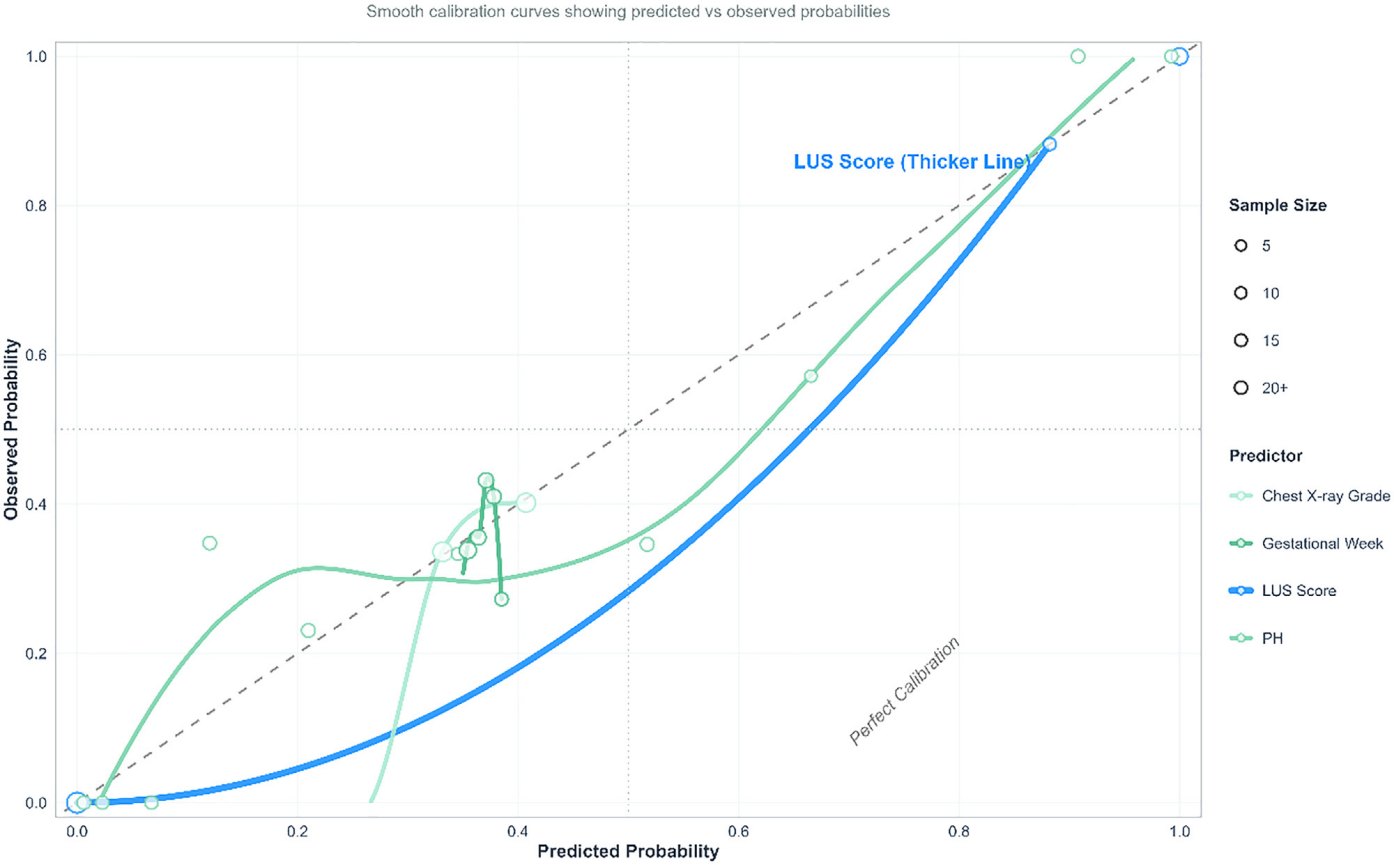
Calibration curves. LUS score demonstrates optimal calibration: the calibration curve for the LUS score (thick blue line) lies closest to the ideal reference line, indicating the best agreement between predicted and observed probabilities across the entire probability range.

Subgroup analyses, interactions, and robustness checks: Prespecified subgroups assessed robustness of LUS diagnostic performance: ventilation mode (IMV vs. NIV), gestational age (≤ 39 vs. > 39 weeks), and PPHN status. Heterogeneity in AUC across subgroups and interaction P-values were evaluated using the Cochran–Mantel–Haenszel framework (Table 6).

**Table 6:** Subgroup analysis of LUS score diagnostic performance.

Subgroup	Sample Size	AUC	95 % CI	Sensitivity*	Specificity*	Youden Index	Optimal Threshold
Overall Population	218	0.999	0.997–1	1	0.986	0.986	5.5
Ventilation: Invasive Ventilation	134	0.997	0.992–1	1	0.965	0.965	5.5
Ventilation: Non-invasive Ventilation	84	1.000	1–1	1	1.000	1.000	5.5
LUS Group: LUS ≥6	82	0.906	0.863–0.949	1	0.000	0.812	6.5
Gestational Age: 37–39 weeks	113	0.998	0.995–1	1	0.973	0.973	5.5
Gestational Age: 40–42 weeks	105	1.000	1–1	1	1.000	1.000	5.5
PPHN: Without PPHN	216	0.999	0.997–1	1	0.986	0.986	5.5

*Sensitivity and specificity calculated at LUS threshold of 6.

Youden Index = Sensitivity + Specificity - 1.

Total subgroups analyzed: 7.

• Subgroup analysis tests LUS score robustness across different patient groups.

• Four predefined subgroups: ventilation type, LUS cutoff, gestational age, PPHN.

• LUS score shows consistent diagnostic performance across all subgroups.

• Results support the generalizability of LUS for PS treatment prediction.

• Analysis excludes groups with insufficient data or homogeneous outcomes.

All statistical code and datasets will be made publicly available for verification.

## Results

### Baseline characteristics

A total of 218 infants with confirmed MAS were included and stratified by receipt of surfactant therapy into a surfactant group (*n* = 80) and a non-PS group (*n* = 138). Demographic and clinical baseline characteristics are summarized in Table 1. Demographics: There were no significant differences in sex distribution (*p* = 1.00) or gestational age (median: 39 weeks in the non-surfactant group vs. 40 weeks in the surfactant group, *p* = 0.704). Clinical indices: The surfactant group had significantly higher LUS scores (median: 8.5 vs. 4, *p* < 0.001), higher fraction of inspired oxygen (FiO₂; median: 50 % vs. 30 %, *p* < 0.001), higher oxygenation index (OI; median: 10 vs. 7, *p* < 0.001), and lower pH (median: 7.08 vs. 7.21, *p* < 0.001).

Complications and length of stay: Length of stay was longer in the surfactant group (median: 8 vs. 6 days, *p* < 0.001). Chest X-ray grade distribution (*p* = 0.413) and the incidence of complications such as PPHN (*p* = 0.134) did not differ significantly between groups.

### Predictive performance of LUS and other indices for surfactant requirement

Receiver operating characteristic (ROC) analyses were used to evaluate the prediction of PS requirement (Table 2). LUS score: At the optimal cutoff of 6, the AUC was 0.999 (95 % CI, 0.997–1) with 100 % sensitivity, 98.6 % specificity, and 93.1 % accuracy. Its AUC was significantly higher than that of pH (AUC = 0.906, *p* < 0.001) and chest X-ray grade (AUC = 0.539, *p* < 0.001).

Comparison with conventional indices: Although OI and FiO₂ guided surfactant decisions in this study, the predictive performance of LUS (AUC = 0.999) numerically exceeded OI (AUC = 0.9673) and FiO₂ (AUC = 0.9527). DeLong tests showed significant AUC differences between LUS and OI (*p* = 0.021) and between LUS and FiO₂ (*p* = 0.015).

### Multivariable logistic regression

To control for potential confounding, the authors fitted multivariable logistic regression models (Table 3) to assess the independent association between LUS score and surfactant use. Model performance: Across three handling schemes (clinical grouping, winsorization, and L1 regularization), model AUCs all exceeded 0.98, indicating excellent discrimination. Key finding: After adjustment for gestational age, sex, FiO₂, chest X-ray grade, and pH, the LUS score remained a strong independent predictor of surfactant use (Scheme C: OR = 2.73, 95 % CI: 1.00–3.81). Other covariates (e.g., gestational age, sex, FiO₂) did not show significant independent associations in the final model.

### Model calibration and clinical utility

Decision curve analysis (DCA) (Table 4): Within clinically relevant threshold probabilities of 10 %–40 %, an LUS-guided strategy provided higher net benefit than “treat-all,” “treat-none,” “pH only,” or “pH plus chest X-ray” strategies. For example, at a threshold probability of 0.3, the net benefit for the LUS strategy was 0.363, superior to alternatives.

Calibration (Table 5): The LUS-based model showed the lowest calibration error (0.0162) and a Brier score of 0.0081, outperforming chest X-ray grade (calibration error: 0.4617), pH (0.2259), and gestational age (0.4644), indicating excellent agreement between predicted and observed probabilities.

### Subgroup analyses

Subgroup analyses assessed robustness across clinical strata (Table 6). Ventilation mode: LUS demonstrated excellent and consistent performance in both the invasive mechanical ventilation group (*n* = 134, AUC = 0.997) and the noninvasive ventilation group (*n* = 84, AUC = 1.000). Gestational age strata: Predictive ability was comparable between 37 and 39 weeks (*n* = 113, AUC = 0.998) and 40–42 weeks (*n* = 105, AUC = 1.000). Complication status: Among infants without PPHN (*n* = 216), LUS maintained very high accuracy (AUC = 0.999). Conclusion: The predictive value of LUS for surfactant requirement is robust across patient subgroups, supporting broad clinical applicability.

### Distribution of LUS scores and probability of surfactant use

**Figure 6:**
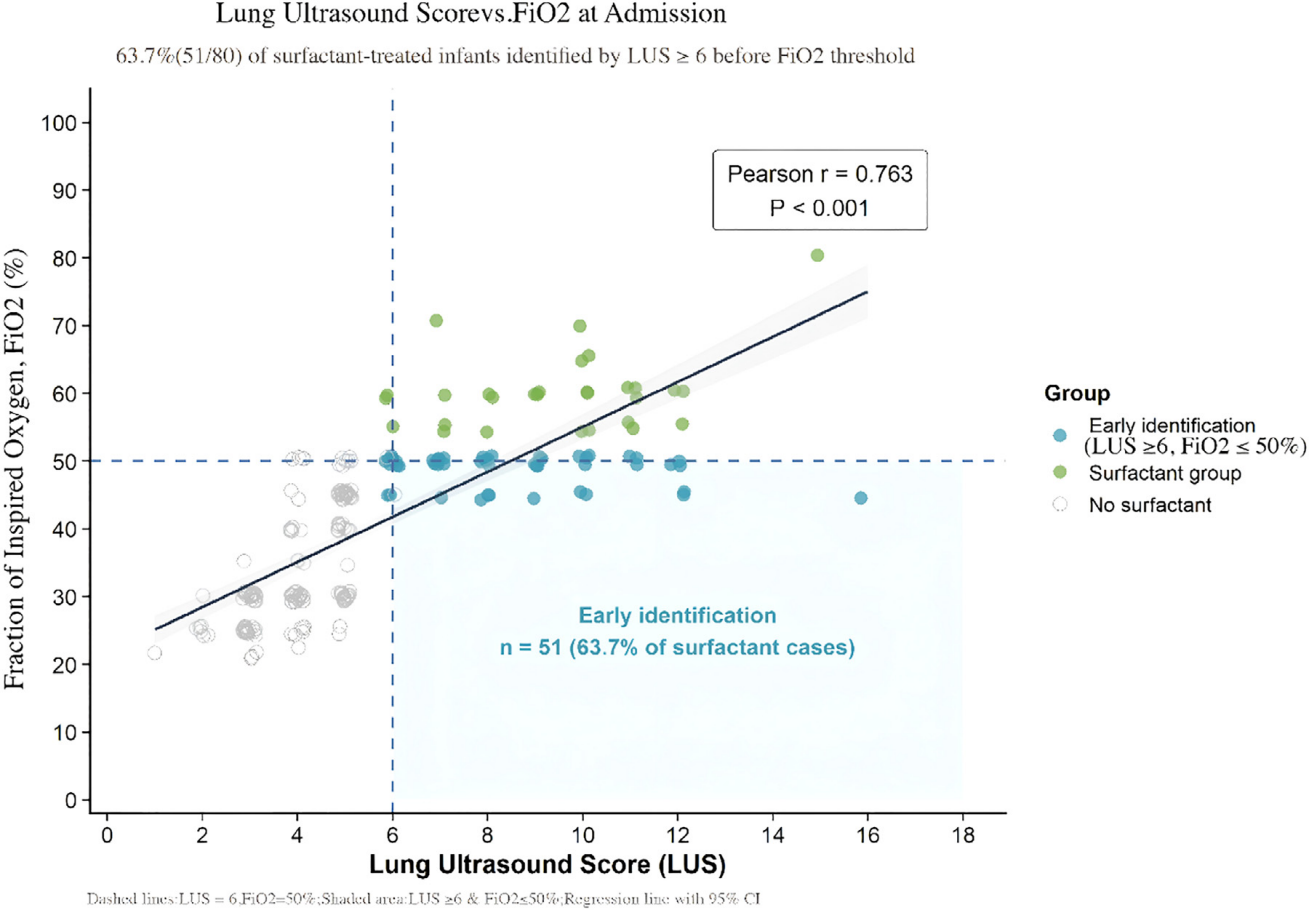
Scatter plot. Strong positive correlation: The LUS score demonstrated a significant strong positive correlation with FiO₂ (*r* = 0.763, *p* < 0.001), indicating that LUS effectively reflects the oxygenation requirements of neonates. - Early Identification Efficacy: Among infants who received surfactant therapy, 63.7 % could be identified early (LUS ≥ 6) before their FiO₂ requirement reached 50 %, providing a basis for timely intervention.

Score distribution: LUS scores were concentrated at higher values in the surfactant group (median 8.5) and at lower values in the non-surfactant group (median 4), with a significant distributional difference (*p* < 0.001). Probability prediction: Restricted cubic spline (RCS) curves indicated a continuous dose–response relationship between LUS and the probability of surfactant use, with a sharp increase in probability once the LUS score exceeded 6 (Figure 6).

## Discussion

In this prospective multicenter study, the authors found that a lung ultrasound score (LUS score) ≥ 6 obtained within 30 min of admission demonstrated near-perfect accuracy (AUC 0.999) in predicting the need for surfactant therapy in neonates with MAS. Furthermore, this result remained consistent across key subgroups, including different gestational ages and respiratory support modes. This suggests that lung ultrasound can serve as an effective early decision-support tool in the management of MAS.

The strong predictive value of lung ultrasound may stem from its ability to directly visualize the complex pulmonary pathological changes associated with MAS. Unlike RDS, which is characterized by a diffuse distribution of surfactant deficiency, MAS involves the combined effects of airway obstruction, inflammatory injury, and surfactant inactivation [21]. Our findings are consistent with previous studies validating the predictive value of lung ultrasound in preterm infants with RDS [9], but further extend its scope of application. Although the pathological changes in MAS are more complex and heterogeneously distributed, the LUS score still exhibited extremely high predictive accuracy (AUC 0.999), which is comparable to, or even slightly higher than, the AUC values reported in RDS studies (0.90–0.98).

This finding has significant clinical implications: in MAS, lung ultrasound not only captures the "surfactant inactivation" component but also integrates the mechanical obstruction and inflammatory response of the disease, providing an integrated morphological assessment of multifocal lung injury. The primary clinical significance of this study lies in its potential to standardize and accelerate treatment decisions for MAS. By providing an immediate morphological assessment at admission, lung ultrasound can identify infants who will eventually require surfactant hours before they meet traditional oxygenation criteria (Oxygenation Index > 8 or FiO_2_ > 0.5). This time window may allow for earlier intervention, thereby mitigating lung injury and shortening the duration of mechanical ventilation, facilitating a paradigm shift from reacting to physiological deterioration to proactively intervening in pulmonary pathology.

This study has several limitations. First, the accuracy of lung ultrasound remains somewhat operator-dependent, although the authors demonstrated high inter-rater reliability (Fleiss' kappa 0.85). Second, as a purely observational study, our protocol did not include continuous, time-stamped measurements; therefore, the authors cannot definitively prove that lung ultrasound identifies surfactant need earlier than traditional markers. Prospective interventional trials are needed to establish the true temporal advantage. Third, MAS is often complicated by Persistent Pulmonary Hypertension of the Newborn (PPHN), yet the authors did not integrate Targeted Neonatal Echocardiography (TNE) to comprehensively assess cardiopulmonary interactions [22., 23., 24., 25., 26.]. Fourth, while lung aeration heterogeneity varies significantly across different neonatal respiratory diseases, the authors did not calculate the intra-patient coefficient of variation for this cohort, data which could have better reflected the intra-individual distribution characteristics of the disease [27]. As this prospective study was initiated in 2019, current guidelines have since been updated to recommend a 10-zone protocol. Consequently, the authors plan to conduct a large-scale randomized controlled trial in the future based on this 10-zone method.

In summary, a lung ultrasound score ≥ 6 is a highly accurate and robust indicator for predicting the need for surfactant therapy in neonates with meconium aspiration syndrome. Despite the aforementioned limitations, the present study’s results support the incorporation of lung ultrasound into the routine admission assessment protocol for MAS. Future interventional trials are needed to further validate this approach and to refine the predictive capability of this promising technology by combining it with TNE and intra-patient variability indices.

## Conclusion

This study demonstrates that during the early admission period for infants with MAS, a LUS≥ 6 possesses extremely high predictive value for the initiation of pulmonary surfactant therapy (AUC 0.999, sensitivity 100 %, specificity 98.6 %). This non-invasive tool, based on direct morphological assessment of the lung, demonstrates superior predictive accuracy compared to traditional oxygenation parameters and maintains high robustness across various clinical subgroups. The authors recommend incorporating standardized LUS assessment into the routine admission evaluation protocol for infants with MAS, particularly for those requiring respiratory support.

## Authors’ contributions

Qi Chen, Zekai Yu, Wei Xiong, Lei Cao: Formal Analysis, Writing-Original Draft, Writing-Review & Editing. Mei Zheng, Fei Wang, Shasha Wu, Rijin Yu, Minli Zhou, Cheng Guo, Lin Dong, Shuang Liu: Formal Analysis, Writing-Review & Editing.

## Data availability

For access to the raw data, please contact the corresponding author.

## Compliance with ethical standards

This study has been approved by the Ethics Committee of Shangrao Maternal and Child Health Hospital, with the ethical approval number SRFB20231206037, dated December 6, 2023.

This study strictly adheres to the Declaration of Helsinki and relevant ethical guidelines, ensuring the full protection of participants' rights to informed consent, privacy, and data security. All participants signed informed consent forms upon admission, clearly understanding the purpose, procedures, potential risks, and benefits of the study. No unnecessary risks or harm were imposed on participants during the research process.

## Funding declaration

The authors declare that no external funding was received for this work.

## Use of generative AI

No generative AI was used during the preparation of this manuscript.

## Conflicts of interest

The authors declare no conflicts of interest.
